# Study protocol: precision of a protocol for manual intramuscular needle placement checked by passive stretching and relaxing of the target muscle in the lower extremity during BTX-A treatment in children with spastic cerebral palsy, as verified by means of electrical stimulation

**DOI:** 10.1186/1471-2431-13-129

**Published:** 2013-08-22

**Authors:** Jessica Warnink-Kavelaars, Roland Jeroen Vermeulen, Jules Guilhelmus Becher

**Affiliations:** 1 Department of Rehabilitation Medicine, MOVE Research Institute Amsterdam, VU University Medical Centre, Boelelaan 1018, Amsterdam, the Netherlands; 2 Division of Child and Adolescent Rehabilitation, Reade Centre for Rehabilitation and Rheumatology, Overtoom 283, Amsterdam, the Netherlands; 3 Department of Child Neurology, Neuroscience Campus Amsterdam, VU University Medical Centre, Boelelaan 1018, Amsterdam, the Netherlands

**Keywords:** Needle placement, Botulinum toxin type-A, Electrical stimulation, Children, Cerebral palsy, Spasticity, Injections

## Abstract

**Background:**

Intramuscular injection of botulinum toxin type-A given by manual intramuscular needle placement in the lower extremity under general anaesthesia is an established treatment and standard of care in managing spasticity in children with spastic cerebral palsy. Optimal needle placement is essential. However, reports of injection and verification techniques used in previous studies have been partly incomplete and there are methodological shortcomings. This paper describes a detailed protocol for manual intramuscular needle placement checked by passive stretching and relaxing of the target muscle for each individual muscle injection location in the lower extremity during botulinum toxin type-A treatment under general anaesthesia in children with spastic cerebral palsy. It explains the design of a study to verify this protocol, which consists of an injection technique combined with a needle localizing technique, as by means of electrical stimulation to determine its precision.

**Methods:**

Setting: University Medical Centre, Department of Paediatric Rehabilitation Medicine, the Netherlands.

Design: prospective observational study.

Participants: children with spastic cerebral palsy, aged 4 to 18 years, receiving regular botulinum toxin type-A treatment under general anaesthesia to improve their mobility, are recruited from the Department of Paediatric Rehabilitation Medicine at VU University Medical Centre, Amsterdam, the Netherlands.

Method: a detailed protocol for manual intramuscular needle placement checked by passive stretching and relaxing of the target muscle has been developed for each individual muscle injection location of the adductor brevis muscle, adductor longus muscle, gracilis muscle, semimembranosus muscle, semitendinosus muscle, biceps femoris muscle, rectus femoris muscle, gastrocnemius lateralis muscle, gastrocnemius medialis muscle and soleus muscle. This protocol will be verified as by means of electrical stimulation.

Technical details: 25 mm or 50 mm Stimuplex-needle and a Stimuplex-HNS-12 electrical stimulator will be used.

**Discussion:**

Botulinum toxin type-A injected in the intended muscle is expected to yield the greatest effect in terms of activities. Protocols for manual intramuscular needle placement should be described in detail and verified to determine its precision. Detailed and verified protocols are essential to be able to interpret the results of botulinum toxin type-A treatment studies.

## Background

Cerebral palsy (CP) is defined as a disorder of posture and movement due to a defect or lesion in the immature brain [1]. It causes physical and movement disability in childhood with an annual incidence of 2 to 3 per 1000 live born children [1,2]. It is a non-progressive motor disorder. The type of movement disorder is classified as spastic, ataxic, dyskinetic, or a combination. To manage spasticity in children with spastic cerebral palsy botulinum toxin type-A (BTX-A) injected by manual intramuscular needle placement in the lower extremity under general anaesthesia is an established treatment and standard of care in children with spastic CP [3-6].

BTX-A is produced by the bacterium Clostridium botulinum, and blocks the release of acetylcholine at the neuromuscular junction. This chemodenervation causes temporary focal weakness of the injected muscle. Over a period of weeks to months, collateral sprouting of the nerve results in reinnervation of the muscle, which results in recovery of the neuromuscular junctions [7-15].

There are various intramuscular injection techniques and needle localizing techniques to administer BTX-A treatment. Location of the needle after manual intramuscular needle placement can be checked by passive stretching and relaxing of the target muscle (PSRM) [16-21], palpation [18,21] and imaging and guiding tools: electrical stimulation (ES) [16,18,21-23], ultrasound [19-21,23,24], computer tomography, X-ray [14,16,17,19,22,24,25] and electromyography [17,19,22].

PSRM is a rapid intramuscular needle localization technique, useful for larger muscles, especially in the lower extremity, which can be performed by one doctor and one assistant without sophisticated equipment.

The selection of muscles to be injected depends on the limitations the patient experiences in some activities due to abnormal activation and activity of the muscles. Injecting BTX-A at the correct location in the intended muscle is expected to yield the greatest effect in terms of activities. Therefore locating the right muscle is an important part of the treatment. Protocols of intramuscular needle placement and intramuscular needle placement verification techniques should be described in detail and verified to determine its precision. Detailed and verified protocols are essential to be able to interpret results of BTX-A treatment studies.

To evaluate previous research on this topic, the literature was searched from the first relevant article on this topic in 1994 to April 2013 using Pubmed, Cochrane database and references of related articles. Keywords used were needle placement, botulinum toxin type-A, cerebral palsy, muscle, electrical stimulation, children, spasticity, injections, echo and ultrasound. MeSH headings used were botulinum toxin type-A, needle, cerebral palsy, muscle spasticity, children, injections, muscle. All languages were included.

In a recent randomised controlled trial, two botulinum toxin injection techniques were compared on the functional improvement of the leg of children with CP. The efficacy of BTX-A treatment was compared between intramuscular injections guided by electrical stimulation and intramuscular injections guided by palpation. The treatment groups received two weeks of physiotherapy after BTX-A treatment. A third group received physiotherapy without BTX-A treatment. The paper reported that intramuscular injection of BTX-A guided by electrical stimulation plus physiotherapy was likely to be best in terms of improving functional performance and spasticity [18]. A similar study compared “free hand” intramuscular needle placement guided by anatomic landmarks, palpation and passively stretching of the muscles with intramuscular needle placement guided by electrical stimulation. “Free hand” manual intramuscular needle placement was found to be acceptable only for the gastrocnemius and soleus muscles (75%) and unacceptable for all the other muscles investigated [16]. A comparative study described intramuscular needle placement guided by ultrasound for the gastrocnemius muscle. The accuracy of the manual needle placement into the medial head was found to be satisfactory, but the accuracy of the lateral head placement was disappointing [21].

The present study describes a detailed protocol for manual intramuscular needle placement in the lower extremity checked by PSRM for each individual muscle injection location during botulinum toxin type-A treatment under general anaesthesia in children with spastic cerebral palsy (see Additional file 1). It explains the design of a study to verify this protocol, which consists of an injection technique combined with a needle localizing technique, as by means of ES and calculates its precision.

## Methods/Design

### Methods

A detailed protocol for manual intramuscular needle placement checked by PSRM has been developed for each individual muscle and its injection locations of the adductor brevis muscle (ADB), adductor longus muscle (ADL), gracilis muscle (GR), semimembranosus muscle (SEM), semitendinosus muscle (SET), biceps femoris muscle (BF), rectus femoris muscle (RF), gastrocnemius lateralis muscle (GL), gastrocnemius medialis muscle (GM) and soleus muscle (SO) (see Additional file 1). This protocol describes the origin, the insertion, the relationship to other structures, the innervation and function of the muscle. It explains the start position of the patient at physical examination for injection, how to support and fixate the leg and the skills to localise the muscle belly and the different injection locations of the target muscle. It clarifies the direction of the needle, the intramuscular needle placement technique and explains the way to check the correct intramuscular needle location by PSRM. It also shows needle placement hazards for each muscle separately.

This protocol, which consists of a manual intramuscular needle placement technique combined with a needle localizing technique, will be verified as by means of ES to determine its precision.

### Design

This is a prospective observational study.

Approval was obtained from the Medical Research Ethics Committees of the Medical Centre, Amsterdam, the Netherlands and MOVE Research Institute Amsterdam of the VU University Medical Centre, Amsterdam, the Netherlands. This study is in compliance with the Helsinki Declaration. The research will be conducted according to the 2004 version of the principles of the Declaration of Helsinki and in accordance with the Dutch Medical Research Involving Human Subjects Act (WMO). Risks associated with participation and physical discomfort are very small to negligible, and participation is not associated with greater physiological discomfort compared to regular BTX-A treatment [9,11,26-30].

### Setting

University Medical Centre, Department of Paediatric Rehabilitation Medicine, the Netherlands.

### Participants

Children with spastic cerebral palsy, aged 4 to 18 years, receiving regular BTX-A treatment under general anaesthesia to improve their mobility, will be recruited from the Department of Paediatric Rehabilitation Medicine, VU University Medical Centre, Amsterdam, the Netherlands.

Inclusion criteria:

1. Children aged 4 to 18 years

2. Diagnosis of spastic CP

3. Planned for regular BTX-A treatment under general anaesthesia

4. Informed consent from caregivers and child if capable

Exclusion criteria:

1. Risk > 2 of the American Society of Anesthesiologists (ASA) physical status classification system during anaesthesia

2. Previous surgery of the muscles included in the study

3. Concomitant muscle-related diseases

4. Infection at the location of needle placement

### Sample size calculation

Each individual muscle injection location of the ADB, ADL, GR, SEM, SET, BF, RF, GL, GM and SO after manual intramuscular needle placement checked by PSRM will be investigated by ES to determine correct needle placement.

The positive predictive value (PPV) or precision is clinically the most relevant parameter to assess the value of this manual intramuscular needle placement checked by PSRM, as it indicates the proportion of the true positive needle placements in the intended muscle against all, true and false, positive needle placements in the intended muscle. If we assume that 90% of the test outcome of manual intramuscular needle placements observed after PSRM is determined to be positive, with α = 0.05 and a power of 80%, then 41 observations of a positive manual intramuscular needle placement will be required for the injection location for each individual muscle. If we assume that approximately 70% of the test outcome of manual intramuscular needle placements observed after PSRM is determined to be positive, with α = 0.05 and a power of 80%, then a total of 60 observations of a positive manual intramuscular needle placement will be required for the injection location for each individual muscle.

### Recruitment and consent

Patients with spastic CP aged 4 to 18 years, of both sexes, treated at the Department of Paediatric Rehabilitation Medicine, VU University Medical Centre, Amsterdam, the Netherlands, will be recruited by their own doctor during a consultation about regular BTX-A treatment. The caregivers and the children will be given information about the research project. A patient information letter, an explanation of the study by means of a cartoon, information about insurance issues, an informed consent form and an envelope to return the informed consent form will be handed to the caregivers at the end of the consultation. The participants will be given a telephone appointment at least 48 hours before the BTX-A treatment, to answer any questions they may have.

### Study procedures

All children will receive their planned BTX-A treatment under general anaesthesia in the operating rooms of the VU University Medical Centre. The protocol for manual intramuscular needle placement checked by PSRM will be used (see Additional file 1).

**Figure 1:**
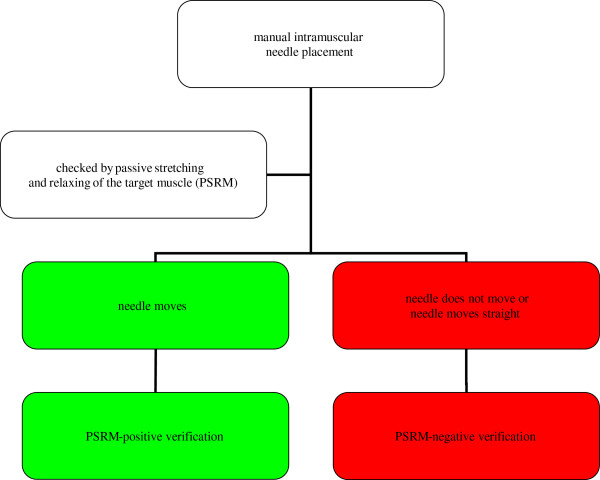
**Flowchart for the protocol for manual intramuscular needle placement checked by PSRM.** Manual intramuscular needle placement checked by PSRM will be assessed as a PSRM-positive verification when the needle moves upon PSRM. Manual intramuscular needle placement will be defined as a PSRM-negative verification when there is no movement or only a small straight movement of the needle upon PSRM.

A 25 mm or 50 mm Stimuplex needle will be used for the injection. Manual intramuscular needle placement will be assessed as a PSRM-positive verification when the needle moves upon passive stretching and relaxing of the intended muscle. Manual intramuscular needle placement will be defined as a PSRM-negative verification when there is no movement or only a small straight movement of the needle upon passive stretching and relaxing of the muscle (see Figure 1). Whether or not the needle is positioned correctly, it will at this stage not be removed or repositioned.

**Figure 2:**
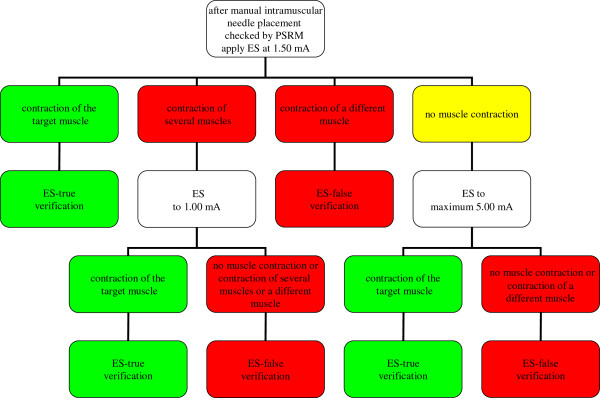
**Flowchart for the protocol for manual intramuscular needle placement checked by PSRM as verified by means of ES.** Whether or not the needle is positioned correctly after manual intramuscular needle placement checked by PSRM; the needle at this stage will not be removed or repositioned. ES will be initiated at 1.50 mA. A palpable and visible contraction of only the target muscle will be assessed as an ES-true verification. The ES level will be reduced to 1.00 mA if more than one muscle contracts at the same time. If only the target muscle shows a palpable and visible contraction after the ES level is reduced, the needle location will be defined as an ES-true verification. Contraction of a different muscle will be assessed as ES-false verification. When there is no contraction at all, the current will be increased to a maximum of 5.0 mA. If there is still no muscle contraction or a different muscle contracts, or many muscles contract at the same time, this will be defined as an ES-false verification.

The needle location will then be verified by means of a Stimuplex HNS 12 electrical stimulator. ES will be initiated at 1.50 mA. A palpable and visible contraction of only the target muscle will be assessed as an ES-true verification. The ES level will be reduced to 1.00 mA if more than one muscle contracts at the same time. If only the target muscle shows a palpable and visible contraction after the ES level is reduced, the needle location will be defined as an ES-true verification. Contraction of a different muscle will be assessed as ES-false verification. When there is no contraction at all, the current will be increased to a maximum of 5.0 mA. If there is still no muscle contraction or a different muscle contracts, or many muscles contract at the same time, this will be defined as an ES-false verification. A palpable and visible contraction of only the target muscle is defined as an ES-true verification (see Figure 2). After this procedure, regular BTX-A treatment will be continued and the needle can be readjusted if necessary.

All data will remain anonymous, to everyone except the principal investigator.

### Statistical analysis

Analyses will be performed using the Statistical Package for the Social Sciences, version 15.0 (SPSS, IBM, New York, USA). The main object of the analysis will be the contingent table of manual intramuscular needle placement checked by PSRM observations versus the corresponding verification as by means of ES observations (see Table 1). This table will be used to calculate the positive predictive value (PPV), with its confidence interval of 95%, as the main objective for each individual muscle and muscle injection location. Positive predictive value or precision is defined as the proportion of the PSRM true positive observations against all the positive observations, both PSRM true positive and PSRM observations. A PPV of 80% with 95% confidence interval and a lower bound of 70% is defined as a minimal acceptable proportion. In addition, negative predictive value (NPV), sensitivity and specificity will also be computed, with their confidence intervals of 95%.

**Table 1:** Contingent table of manual intramuscular needle placement checked by PSRM observations versus the corresponding observations with ES

		**Electrical stimulation**		
		**ES-true**	**ES-false**	
**Protocol for manual intramuscular needle placement checked by PSRM**	PSRM-positive	*True positive*	*False positive*	**PPV or precision**
	PSRM-negative	*True negative*	*False negative*	NNP
		Sensitivity	Specificity	Accuracy

This table will be used to calculate the positive predictive value (PPV), with its confidence interval of 95%, as the main objective for each individual muscle and muscle injection location. The positive predictive value or precision is defined as the proportion of true positive results against all the positive results, both true positives and false positives. In addition, negative predictive value (NPV), sensitivity and specificity will also be computed, with their confidence intervals of 95%.

The measures of association (odds ratios) will be compared with Cochran’s test for homogeneity. Factors which might be related to a false positive intramuscular needle placement checked by PSRM, such as type of target muscle, injection location in the different target muscles, left and right legs, gender, height, weight, body mass index, age and level of experience of the doctor will be assessed by means of a multifactorial analysis of variance.

## Discussion

Botulinum toxin type-A injected in the intended muscle is expected to yield the greatest effect in terms of activities. Protocols for manual intramuscular needle placement should be described in detail and verified to determine the precision of the injection method. Detailed and verified protocols are essential to be able to interpret the results of botulinum toxin type-A treatment studies.

Three relevant articles reporting specific on intramuscular needle placement in live humans failed to provide detailed descriptions of a protocol for intramuscular injection for each individual muscle injection location [16,18,21].

In a randomized control trial two BTX-A intramuscular injection techniques were compared on the functional improvement of the leg of children with CP. The efficacy of BTX-A treatment was compared between intramuscular injections guided by electrical stimulation and intramuscular injections guided by palpation. The statistical analysis of all intramuscular injections of one group were merged [18]. According to the International Classification of Functioning, Disability and Health for Children and Youth (ICF-CY) spasticity of the muscle (body structure) can impair function, which can cause limitations of activities and participation in daily life. There are many factors as health condition, environmental and personal factors which also influence these functional, activity and participation outcomes. Clinical outcomes are blurred by all the factors described above so this methodological choice obscures the results. When there is no clear description of a detailed and verified protocol for intramuscular needle placement merging of intramuscular injections during analysis is not acceptable.

In an other article the author merged data of manual intramuscular needle placements of the soleus muscle and the gastrocnemius muscle and concluded that the percentage of true positive manual needle placements out of all positive needle placements checked by ES was acceptable [16]. Combining muscles and muscle locations does not provide any useful information and does not allow making conclusions about precision or accuracy of manual intramuscular needle placement and verification techniques.

Clarifying statistical terms used in a manuscript is of great importance to make a clear interpretation of the results. In this study protocol the main object of analysis will be the contingent table of intramuscular needle placement checked by PSRM observations versus the corresponding observations with ES (see Table 1). This table will be used to calculate the precision or PPV which is defined as the proportion of true positive needle placements against all positive needle placement results (both true positives and false positives). During BTX-A treatment, BTX-A should be injected only after manual intramuscular needle placement checked positive by PSRM, ES or echo using a verified protocol.

In this study protocol the statistical term accuracy is defined as the proportion of true needle placements (both true positives and true negatives). In a similar article the authors calculated the accuracy which was defined differently as the proportion of positive needle placements checked by ES out of all manual intramuscular injections. Negative manual intramuscular needle placements were not investigated and not checked by ES [16]. One article described intramuscular needle placement based on only landmarks and palpation and in a randomized clinical trial needle placement was based on palpation of the muscle belly after increasing muscle tone by stretching the spastic ankle plantar flexors. Both studies did no use a PSRM technique or an other verification technique to localize the correct needle placement in the target muscle [18,21]. The three methodological methods used do not allow the construction of a contingent table of intramuscular needle placements checked by a needle location technique observations versus observations verified as by means of ES or echo. Nor do they allow the calculation of positive predictive values or precision with their confidence intervals as the main objective for each individual muscle and muscle injection location neither the calculation of the negative predictive value, sensitivity and specificity. This obscures the outcome and allows no clear answer to the question whether intramuscular needle placement is precise for a specific target muscle and a specific injection location.

Additional file 1 provides no guidance of manual intramuscular needle placement depth because it is complicated. The recruited children are aged between 4 and 18 years old and needle depth might depend on the child, age, muscle type, muscle injection location, left and right legs, gender, height, weight and body mass index.

This protocol for manual intramuscular needle placement checked by PSRM is described in detail. If verification of this protocol as by means of ES shows a minimal precision or PPV of 80% with 95% confidence interval and a lower bound of 70% then this protocol is precise and suitable for use in clinical practice of BTX-A treatment in children with spastic cerebral palsy in the lower extremity.

## Abbreviations

ADB: Adductor brevis muscle; ADL: Adductor longus muscle; ASA: American society of anesthesiologists; BF: Biceps femoris muscle; BMI: Body mass index; BTX-A: Botulinum toxin type-A; CP: Cerebral palsy; ES: Electrical stimulation; GL: Gastrocnemius lateralis muscle; GM: Gastrocnemius medialis muscle; GR: Gracilis muscle; ICF-CY: International classification of functioning, disability and health for children and youth; METC: Medical research ethics committee; NPV: Negative predictive value; PPV: Positive predictive value; PSRM: Passive stretching and relaxing of the target muscle; RF: Rectus femoris muscle; SEM: Semimembranosus muscle; SET: Semitendinosus muscle; SO: Soleus muscle; WMO: Dutch medical research involving human subjects act.

## Competing interests

The authors declare that they have no competing interests.

## Pre-publication history

The pre-publication history for this paper can be accessed here:

http://www.biomedcentral.com/1471-2431/13/129/prepub

## Supplementary Material

Additional file 1 **Descriptive protocol for manual intramuscular needle placement checked by passive stretching and relaxing of the target muscle.** This file describes a detailed protocol for manual intramuscular needle placement checked by passive stretching and relaxing of the target muscle (PSRM) to determine correct needle placement for each individual muscle injection location during botulinum toxin type-A treatment under general anaesthesia in children with spastic cerebral palsy for the adductor brevis muscle, adductor longus muscle, gracilis muscle, semimembranosus muscle, semitendinosus muscle, biceps femoris muscle, rectus femoris muscle, gastrocnemius lateralis muscle, gastrocnemius medialis muscle and soleus muscle. Manual intramuscular needle placement will be assessed as a PSRM-positive verification when the needle moves upon passive stretching and relaxing of the intended muscle. Manual intramuscular needle placement will be defined as a PSRM-negative verification when there is no movement or only a small straight movement of the needle upon passive stretching and relaxing of the muscle. For each muscle separately this protocol describes the origin, the insertion, the relationship to other structures, the innervation and function of the muscle. It explains the start position of the patient at physical examination for injection, how to support and fixate the leg and the skills to localise the muscle belly and the different injection locations of the target muscle. It clarifies the direction of the needle, the intramuscular needle placement technique and explains the way to check the correct intramuscular needle location by passive stretching and relaxing of the target muscle. It also shows needle placement hazards for each muscle separately. Click here for file
