# Supplementary material for: Study protocol: precision of a protocol for manual intramuscular needle placement checked by passive stretching and relaxing of the target muscle in the lower extremity during BTX-A treatment in children with spastic cerebral palsy, as verified by means of electrical stimulation
Source: BMC Pediatr. 2013 Aug 22;13:129. doi: 10.1186/1471-2431-13-129 (PMC3765895; doi:10.1186/1471-2431-13-129)
Supplement: Additional file 1 — Descriptive protocol for manual intramuscular needle placement checked by passive stretching and relaxing of the target muscle. This file describes a detailed protocol for manual intramuscular needle placement checked by passive stretching and relaxing of the target muscle (PSRM) to determine correct needle placement for each individual muscle injection location during botulinum toxin type-A treatment under general anaesthesia in children with spastic cerebral palsy for the adductor brevis muscle, adductor longus muscle, gracilis muscle, semimembranosus muscle, semitendinosus muscle, biceps femoris muscle, rectus femoris muscle, gastrocnemius lateralis muscle, gastrocnemius medialis muscle and soleus muscle. Manual intramuscular needle placement will be assessed as a PSRM-positive verification when the needle moves upon passive stretching and relaxing of the intended muscle. Manual intramuscular needle placement will be defined as a PSRM-negative verification when there is no movement or only a small straight movement of the needle upon passive stretching and relaxing of the muscle. For each muscle separately this protocol describes the origin, the insertion, the relationship to other structures, the innervation and function of the muscle. It explains the start position of the patient at physical examination for injection, how to support and fixate the leg and the skills to localise the muscle belly and the different injection locations of the target muscle. It clarifies the direction of the needle, the intramuscular needle placement technique and explains the way to check the correct intramuscular needle location by passive stretching and relaxing of the target muscle. It also shows needle placement hazards for each muscle separately. [file 1471-2431-13-129-S1.pdf]

Additional file 1:

Descriptive protocol for manual intramuscular needle placement checked by passive stretching and relaxing of the target muscle

This additional file is part of the study protocol:

"Precision of a protocol for manual intramuscular needle placement checked by passive stretching and relaxing of the target muscle in the lower extremity during BTX-A treatment in children with spastic cerebral palsy, as verified by means of electrical stimulation"

Jessica Warnink-Kavelaars[1,2]*, Roland Jeroen Vermeulen[3], Jules Guilhelmus Becher[1]
[1] Department of Rehabilitation Medicine, MOVE Research Institute Amsterdam, VU University Medical Centre, Boelelaan 1018, Amsterdam, the Netherlands
[2] Division of Child and Adolescent Rehabilitation, Reade Centre for Rehabilitation and Rheumatology, Overtoom 283, Amsterdam, the Netherlands
[3] Department of Child Neurology, Neuroscience Campus Amsterdam, VU University Medical Centre, Boelelaan 1018, Amsterdam, the Netherlands
* Corresponding author

This file describes a detailed protocol for manual intramuscular needle placement checked by passive stretching and relaxing of the target muscle (PSRM) to determine correct needle placement for each individual muscle injection location during botulinum toxin type-A treatment under general anaesthesia in children with spastic cerebral palsy for the adductor brevis muscle, adductor longus muscle, gracilis muscle, semimembranosus muscle, semitendinosus muscle, biceps femoris muscle, rectus femoris muscle, gastrocnemius lateralis muscle, gastrocnemius medialis muscle and soleus muscle. Manual intramuscular needle placement will be assessed as a PSRM-positive verification when the needle moves upon passive stretching and relaxing of the intended muscle. Manual intramuscular needle placement will be defined as a PSRM-negative verification when there is no movement or only a small straight movement of the needle upon passive stretching and relaxing of the muscle.

For each muscle separately this protocol describes the origin, the insertion, the relationship to other structures, the innervation and function of the muscle. It explains the start position of the patient at physical examination for injection, how to support and fixate the leg and the skills to localise the muscle belly and the different injection locations of the target muscle. It clarifies the direction of the needle, the intramuscular needle placement technique and explains the way to check the correct intramuscular needle location by passive stretching and relaxing of the target muscle. It also shows needle placement hazards for each muscle separately.

The article itself explains the design of a study to verify this protocol, which consists of a manual intramuscular needle placement technique combined with a needle localizing technique, as by means of electrical stimulation to determine its precision. Protocols for manual intramuscular needle placement should be described in detail and verified to determine its precision. Detailed and verified protocols are essential to be able to interpret the results of botulinum toxin type-A treatment studies.

Adductor Brevis Muscle
Origin: a narrow origin of the superior and inferior ramus of the anterior pubis, between the gracilis muscle and the obturator externus muscle [1-4].
Insertion: fibers are inserted by an aponeurosis to the middle third the of the medial lip of the linea aspera of the posterior femur and to the distal two third of the pectinal line.[1-4].
Relationship to other structures: anterior surface: pectineus muscle, adductor longus muscle, obturator artery, obturator vein, obturator nerve; posterior surface: adductor magnus muscle, posterior branches of obturator artery, obturator vein, obturator nerve; lateral surface: obturator externus muscle, iliopsoas muscle; medial surface: gracilis muscle, adductor magnus muscle. Proximally it is pierced by the middle perforating artery [1-4].
Innervation: obturator nerve L2, L3, L4 [1-4].
Function: the adductor brevis muscle adducts and assists in flexion of the hip [1-4]. Depending on the mechanical axis of the femur and the position of the femur to the pelvis the muscle assists in endorotation [1,2] and/or exorotation [1,4] of the hip.
Start position for injection: supine position, the hip is abducted and flexed 90 degrees, the knee is flexed 60 degrees [5,6].
Fixation: the examiner supports the upper leg in abduction with one hand located under the flexed knee. The other hand is free [5].
Joint movement to stretch and relax the muscle: hip abduction and adduction [5,6]. This can be done by the examiner or by a person.
Localisation technique of the muscle belly: palpate with the fingers distal of the anterior ramus of the pubis. A thick string like, triangular in form muscle is felt. It stretches when the hip is abducted. The adductor brevis muscle lies partly posterior to the adductor longus muscle. It's difficult to differentiate from each other [1-5].
Intramuscular needle placement location and direction: place one injection ±2-4 cm distal to the origin. Place the needle 90 degrees to the skin pointing in lateral direction, perforating the adductor longus muscle.
Joint movement to stretch and relax the muscle: hip abduction and adduction [5,6]. This can be done by the examiner or by a person.
Needle placement hazards: perforating/injecting branches of the obturator artery, obturator vein, obturator nerve and wrong muscle [1-4] .

Adductor longus muscle
Origin: ramus superior of the anterior pubis, just inferior to the pubic tubercle [1-4].
Insertion: the fibers extend into the posterior wall of Hunter's canal and attach to the middle third of the medial lip of the linea aspera on the posterior femur [1-4].
Relationship to other structures: anterior surface: fascia lata, femoral artery and vein; posterior surface: adductor brevis muscle, adductor magnus muscle, anterior branches of the obturator artery, vein, and nerves, profunda artery and vein; medial surface: gracilis muscle; lateral surface: pectineus muscle [1-4].
Innervation: obturator nerve L2, L3, L4 [1-4].
Function: the adductor longus muscle adducts and assists in hip flexion [1-4]. Depending on the mechanical axis of the femur and the position of the femur to the pelvis the muscle assists in endorotation [1,2] and/or exorotation [1,4] of the hip.
Start position for injection: supine position, the hip is abducted and flexed 60 degrees and the knee is flexed 60 degrees [5,6].
Fixation: the examiner supports the upper leg in abduction with one hand located under the flexed knee. The other hand is free [5].
Localisation technique of the muscle belly: palpate with the fingers distal of the anterior pubis. A thick string like, triangular in form muscle is felt. It stretches when the hip is abducted. The

adductor brevis muscle lies partly posterior to the adductor longus muscle. It’s difficult to differentiate from each other [1-5].
Intramuscular needle placement location and direction: place one injection ±4-6 cm distal of the anterior pubis. Direct the needle superficially with an angle of 90 degrees to the skin, pointing in posterior direction.
Joint movement to stretch and relax the muscle: hip abduction and adduction [5,6]. This can be done by the examiner or by a person.
Needle placement hazards: perforating/injecting of the femoral artery and vein, obturator artery, vein, and nerves and wrong muscle [1-4].

Gracilis muscle
Origin: a thin aponeurosis from the anterior symphysis pubis and the medial margin of the inferior ramus [1-4].
Insertion: medial proximal tibia, pes anserinus superficialis[1-4].
Relationship to other structures: anterior surface: skin; posterior surface: adductor magnus muscle, adductor longus muscle; lateral surface: adductor magnus muscle, adductor longus muscle; medial surface: skin [1-4].
Innervation: obturator nerve L2, L3, L4 [1-4]
Function: the gracilis muscle is a biarticular muscle and adducts, flexes [1-4] and endorotates [1,2] the hip and assists in flexion and endorotation of the knee [4]. If the lower legs are fixed it helps to maintain the body in an erect posture and assists in anterior tilt of the pelvis [1].
Start position for injection: supine position, the hip is abducted, exorotated and flexed 90 degrees, the knee is flexed 90 degrees [5,6].
Fixation: the examiner supports the upper leg in abduction, exorotation and flexion. The knee rests against the chest of the examiner. One hand supports the lower leg to flex and extent the knee. The other hand is free [5].
Localisation technique of the muscle belly: the gracilis muscle is a long, straplike muscle which fibers run vertically down. It can be felt at the medial condyl of the femur as a rounded tendon, lying most medial. The tendon becomes flattened below the medial condyl of the tibia. From it’s insertion the tendon can be palpated by the fingers in proximal direction. By flexing and extending the knee during palpation the muscle will relax and stretch [1-5].
Intramuscular needle placement location and direction: place the first injection superficially with an angle of 90 degrees to the skin approximately 10%-40% of the origin, pointing in lateral direction. Place the second injection superficially with an angle of 90 degrees to the skin, ±4 cm distal of the first injection, pointing in lateral direction.
Joint movement to stretch and relax the muscle: knee flexion and extension [5,6]. This can be done by the examiner or by a person.
Needle placement hazards: perforating/injecting the saphenous nerve and vein and wrong muscle [1-4].

Semitendinosus muscle
Origin: the tendon is shared with the origin of the biceps femoris muscle at the ischial tuberosity [1-4].
Insertion: it’s a superficial tendon. It inserts to the proximal, medial condyl of the tibia, so called pes anserinus, behind the tendon of the sartorius muscle and below the tendon of the gracilis muscle [1-4].
Relationship to other structures: anterior surface: adductor magnus muscle, femoral artery and vein, sciatic nerve; posterior surface: gluteus maximus muscle; lateral surface: biceps femoris muscle; medial surface: gracilis muscle, adductor magnus muscle [1-4].
Innervation: tibial nerve, L4 L5 S1 S2 S3 [1-4].

Function: The semitendinosus muscle is a biarticular muscle. It extends the hip, flexes the knee [1-4], endorotates the tibia on the femur when the knee is flexed [1-4] and endorotates the femur when the hip is extended [1,3].
Start position for injection: supine position, the hip is neutral with no adduction or abduction and flexed 90 degrees, the knee is flexed 90 degrees.
Fixation: the examiner supports the upper leg in hip flexion 90 degrees. The knee rests against the chest of the examiner. One hand supports the lower leg to flex and extent the knee. The other hand is free [5].
Localisation technique of the muscle belly: it's a small bandlike muscle. Palpate the muscle at the ischial tuberosity on the medial side deeply through the gluteus maximus muscle. Lateral to the semitendinosus muscle the aponeurosis of the biceps femoris muscle is located. The muscle is stretched and best felt when the knee is extended, alternate knee flexion and extention. Abduction and adduction in the hip differentiates between the gracilis muscle and semitendinosus muscle [1-5].
Intramuscular needle placement location and direction: place the first injection at the middle of the femur, place the second injection approximately ±4 cm distal to the first injection. The cordlike tendon of the semitendinosus starts at 2/3 of the femur so do not place the injection to far distally. Direct the needle superficially with an angle of approximately 90 degrees to the skin pointing in anterior and medial direction.
Joint movement to stretch and relax the muscle: knee flexion and extension [5,6]. This can be done by the examiner or by a person.
Needle placement hazards: perforating/injecting the femoral artery and vein, tibial nerve, sciatic nerve and wrong muscle [1-4].

Semimembranosus muscle
Origin: a thick membranous tendon arises from the ischial tuberosity proximal and lateral of the biceps femoris muscle and the semitendinosus muscle [1-4].
Insertion: posterior part of the medial condyl of the tibia [1-4].
Relationship to other structures: anterior surface: vastus medialis muscle, adductor magnus muscle, sciatic nerve, femoral artery and vein, poplitial artery and vein; posterior surface: semitendinosus muscle; medial surface: gracilis muscle, adductor magnus muscle; lateral surface: semitendinosus muscle.
Innervation: tibial nerve L4,L5,S1,S2 [1-4].
Function: the semimenbranosus muscle is a biarticular muscle. It extends the hip, flexes the knee [1-4], endorotates the tibia on the femur when the knee is flexed [1-4] and endorotates the femur when the hip is extended [1,3].
Start position for injection: supine position, the hip is flexed 90 degrees, the knee is flexed 90 degrees [5,6].
Fixation: the examiner supports the upper leg in abduction, exorotation and flexion. The knee rests against the chest of the examiner. One hand supports the lower leg to flex and extent the knee. The other hand is free [5].
Localisation technique of the muscle belly: The tendon of the gracilis muscle can be felt at the medial condyl of the femur as a rounded tendon, lying most medial. The tendon of the semitendinosus is felt laterally to the tendon of the gracilis muscle. Between these two tendons there is a sulcus in which the muscle belly of the semimembranosus muscle can be palpated. Palpate with the fingers in proximal direction of the knee in the sulcus between the gracilis muscle and semitentinosus muscle. Alternate flexion and extension of the knee joint by stretching the semimembranosus muscle. The muscle belly starts moving under the palpating fingers. To differentiate between the gracilis muscle and semitendinosus muscle

alternate between hip exorotation combined with abduction and endorotation combined with adduction [1-5].
Intramuscular needle placement location and direction: from dorsal view place the first injection on 1/2 of the femur in the sulcus, place the second injection in the sulcus ±4 cm distal of the first injection. Place the needle is with an angle of approximately 70 degrees to the skin, pointing in anterior and lateral direction.
Joint movement to stretch and relax the muscle: knee flexion and extension [5,6]. This can be done by the examiner or by a person.
Needle placement hazards: perforating/injecting the popliteus artery, femoral artery, femoral vein, tibial nerve, sciatic nerve and wrong muscle [1-4].

Biceps femoris muscle
Origin: the muscle has two heads of origin, the long head arises from the ischial tuberosity and distal part of the sacrotuberous ligament, the short head originates between the origin of the adductor magnus muscle and vastus lateralis muscle from the lateral lip of the linea aspera and the lateral supracondylar line of the femur [1-4,7].
Insertion: lateral side of the fibular head [1-4,7].
Relationship to other structures: anterior surface: adductor magnus muscle, sciatic nerve, perforating artery; posterior surface: gluteus maximus muscle; lateral surface: vastus lateralis muscle; medial surface: peroneal nerve, semitendinosus muscle [1-4,7].
Innervation: long head: tibial nerve L5 S1 S2, short head: peroneal nerve L5 S1 S2 [1-4].
Function: the biceps femoris long head muscle is a biarticular muscle which is able to flex and exorotate the knee and assists in hip extension [1-4]. The biceps femoris short head muscle is a monoarticular muscle which flexes and exorotates the knee [1-4].
Start position for injection: supine position, the hip is adducted and flexed 90 degrees, the knee is flexed 90 degrees [5,6].
Fixation: the examiner supports the upper leg in adduction and flexion. One hand supports the lower leg to flex and extent the knee. The other hand is free [5].
Localisation technique of the muscle belly: palpate with the fingers in the poplitial fossa. A cordlike tendon can be felt as a tight ridge. The muscle is felt best when stretched while the knee is extended and the hip endorotated and or adducted. Alternate knee flexion and extention and hip exorotation. The biceps femoris long head muscle passes oblique. Palpate the muscle following the direction from the insertion to the ischial tuberosity. [1-5,7].
Intramuscular needle placement location and direction: place the first injection superficially with an angle of 90 degrees to the skin, pointing in anterior and medial direction ±4 cm from the ischial tuberosity. Place the second injection ±4 cm distal of the first injection approximately ±2 cm lateral of the line from ischial tuberosity and insertion. Place the needle with an angle of 90 degrees to the skin, pointing in anterior and medial direction.
Joint movement to stretch and relax the muscle: knee flexion and extension, hip exorotation and endorotation [5,6]. This can be done by the examiner or by a person.
Needle placement hazards: perforating/injecting the perforating-artery, sciatic nerve and wrong muscle [1-4].

Rectus femoris muscle
Origin: one tendon arises from the anterior inferior iliac spine and the second one originates from the ilium on the upper margin of the acetabulum [1-4].
Insertion: a broad and thick patellar ligament is inserted to the tibial tuberosity, it embraces the patella [1-4].
Relationship to other structures: it’s one of the four quadriceps femoris muscles: rectus femoris muscle, vastus lateralis muscle, vastus medialis muscle and the vastus intermedius

muscle. Anterior surface: satorius muscle; posterior surface: vastus intermedius muscle; lateral surface: vastus lateralis muscle, tensor fascia latae muscle; medial surface: vastus medialis muscle [1-4].
Innervation: femoral nerve L2, L3, L4 [1-4].
Function: the rectus femoris muscle extends the knee and assists in hip flexion [1-4].
Start position for injection: supine position, the hip is abducted in maximal extension and the knee is flexed hanging at the side of the table [5,6].
Fixation: the examiner supports the upper leg in abduction with one hand located just above the knee. The other hand is free [5].
Localisation technique of the muscle belly: Palpate with the fingers proximal of the patella while flexing and extending the knee joint. The muscle is stretched when the knee is flexed. Follow the muscle proximally from the patella to the anterior inferior iliac spine [1-5].
Intramuscular needle placement location and direction: place the first injection approximately 30%-50% distal of the anterior inferior iliac spine, place the second injection at 50-70% of the femur. When botulinum toxin A treatment is given to improve knee extension than place the first injection at 50-70% of the femur and the second injection 70%-90% of the femur. The tendon starts proximally of the patella so do not place the injection too distally. Place the needle superficially with an angle of 90 degrees to the skin.
Joint movement to stretch and relax the muscle: knee flexion and extension [5,6]. This can be done by the examiner or by a person.
Needle placement hazards: perforating the wrong muscle [1-4].

Gastrocnemius medial head muscle
Origin: the medial, larger head originates from the proximal and posterior part of the medial epicondyle of the femur and posterior surface of the femur [1-4].
Insertion: the two heads of the gastrocnemius muscle and the soleus muscle have a common tendon inserted at the posterior surface of the calcaneus, the so called achilles tendon [1-4].
Relationship to other structures: the gastrocnemius muscle is a biarticular muscle and consists of two heads, a lateral and medial head. It’s part of the tripartite muscle referred to as the triceps surae. Anterior surface: soleus muscle, tendon of the plantaris muscle; posterior surface: skin; lateral surface: tibial nerve, poplitial artery and vein, gastrocnemius lateral head muscle; medial surface: saphenus nerve, skin [1-4].
Innervation: tibial nerve S1, S2 [1-4].
Function: the gastrocnemius muscle is a powerful plantar flexor of the foot and flexes the knee [1-4].
Start position for injection: supine position, the hip is flexed 45 degrees and slightly abducted, the knee is extended [5,6].
Fixation: the examiner supports the foot with one hand to keep the leg flexed and abducted with extension of the knee. The leg rests against the chest of the examiner. The other hand is free [5].
Localisation technique of the muscle belly: palpate with the fingers at the medial condyle of the femur while dorsoflexing and plantarflexing the foot. Move distally with the fingers and follow the muscle. The sulcus between the medial and lateral head of the muscle is best felt when the foot is in dorsoflexion. The medial head is located medial of the sulcus. The muscle belly starts from the origin and ends at the middle of the tibia [1-5].
Intramuscular needle placement location and direction: place the first injection approximately 20% of the tibia length distal of the proximal and posterior medial epicondyle of the femur, place the second injection 40-50% distal of the first injection. Place the needle with an angle of 90 degrees to the skin pointing to the anterior direction.

Joint movement to stretch and relax the muscle: foot dorsoflexion and plantarflexion [5,6]. This can be done by the examiner or by a person.
Needle placement hazards: perforating/injecting the poplitial artery and vein, saphenus nerve, tibial nerve and wrong muscle [1-4].

Gastrocnemius lateral head muscle
Origin: the lateral head origins from the proximal and posterior side of the lateral epicondyle of the femur and the posterior surface of the femur [1-4].
Insertion: the two heads of the gastrocnemius muscle and the soleus muscle have a common tendon inserted at the posterior surface of calcaneus, the so called achilles tendon [1-4].
Relationship to other structures: the gastrocnemius muscle is a biarticular muscle and consists of two heads, a lateral and medial head. It's part of the tripartite muscle referred to as the triceps surae. Anterior surface: soleus muscle, plantaris muscle; posterior surface: cutaneus surae lateral nerve, skin; lateral surface: peroneal communis nerve, skin; medial surface: gastrocnemius medial head muscle [1-4].
Innervation: tibial nerve S1, S2 [1-4].
Function: the gastrocnemius muscle is a powerful plantar flexor of the foot and flexes the knee [1-4].
Start position for injection: supine position, the hip is flexed 45 degrees and slightly abducted, the knee is extended [5,6].
Fixation: the examiner supports the foot with one hand to keeps the hip flexed and abducted and the knee in extension. The leg rests against the chest of the examiner. The other hand is free [5].
Localisation technique of the muscle belly: the examiner kneels next to the table. Palpate with the fingers at the lateral condyle of the femur while dorsoflexing and plantarflexing the foot. Move distally with the fingers and follow the muscle. The sulcus between the medial and lateral head of the muscle is felt best when the knee is extended and the foot is dorsoflexed. The lateral head is located ±1-2 cm lateral of the sulcus. The muscle belly starts from the origin and ends at the middle of the tibia [1-5].
Intramuscular needle placement location and direction: place the first injection just distal of the fibular head (20%), place the second injection 40-50% distal of the first injection. Place the needle with an angle of 90 degrees to the skin pointing in the anterior direction.
Joint movement to stretch and relax the muscle: foot dorsoflexion and plantarflexion [5,6]. This can be done by the examiner or by a person.
Needle placement hazards: perforating/injecting cutaneus surae lateral nerve, peroneal communis nerve, and wrong muscle [1-4].

Soleus muscle
Origin: posterior surface of the head of the fibula, proximal 1/3 of the posterior surface of the fibula, soleal line and medial border of the tibia [1-4].
Insertion: the soleus muscle and the two heads of the gastrocnemius muscle have a common tendon inserted at the posterior surface of the calcaneus, the so called achilles tendon [1-4].
Relationship to other structures: the soleus muscle is part of the tripartite muscle reffered to as the triceps surae. Anterior surface: intermuscular fascial septum, tibial nerve, peroneal artery and vein, posterior tibial artery and vein, tibialis posterior muscle, flexor digitorum longus muscle, flexor hallucis longus muscle, peroneal longus muscle, peroneal brevis muscle; posterior surface: plantaris muscle, gastrocnemius medial and lateral head muscle; lateral surface: sureal nerve, saphenus parva vein, skin, medial surface: saphenus magna vein, saphenus nerve, skin [1-4].
Innervation: tibial nerve S1, S2 [1-4].

Function: a powerful plantar flexor of the foot [1-4].
Start position for injection: supine position, the hip is flexed 45 degrees and slightly abducted, the knee is flexed 90 degrees [5,6].
Fixation: the examiner supports the foot with one hand to keep the leg flexed, abducted and the knee extended. The leg rests against the chest of the examiner. The other hand is free [5].
Localisation technique of the muscle belly: palpate with the fingers at the medial condyle of the femur while dorsoflexing and plantarflexing the foot. Move distal with the fingers into the sulcus between the medial and lateral head of the gastocnemius muscle. Keep the fingers into the sulcus at 1/3 of the tibia length, flex the knee 90 degrees and start dorsoflexing and plantarflexing the foot. The soleus muscle slides under the fingers [1-5].
Intramuscular needle placement location and direction: Move with the fingers the medial head of the gastrocnemius muscle to the medial side. Place the first injection with an angle of 70 degrees to the skin pointed to the anterior- medial side of the leg, for the second injection the fingers have to move the lateral head of the gastrocnemius muscle to the anterior side. Direct the needle with an angle of 70 degrees to the skin pointed to the anterior- lateral side of the leg.
Joint movement to stretch and relax the muscle: foot dorsoflexion and plantarflexion [5,6]. This can be done by the examiner or by a person.
Needle placement hazards: perforating/injecting tibial nerve, peroneal artery and vein, posterior tibial artery and vein, sureal nerve, saphenus parva vein, saphenus magna vein, saphenus nerve and the wrong muscle [1-4].

Reference List

1. Gray H, thoroughly revised and re-edited by Warren H.Lewis.: *Anatomy of the Human Body*, 20th edn. Philadelphia: Lea & Febiger, 1918; Bartleby.com, 2000. www.bartleby.com/107/; 1918.

2. Kendall FP, Kendall Mc Creary E: *Muscles: testing and function with posture and pain*, 5th, illustrated edn. Baltimore, United States: Lippincott Williams & Wilkins; 2005.

3. Moore KL: *Clinically oriented anatomy*, 3rd edn. Baltimore, United States: Williams & Wilkins; 1992.

4. Platzer W: *Atlas van de anatomie, bewegingsapparaat, deel 1,* 20 edn. Baarn, the Netherlands, SESAM/HBuitgevers; 2003.

5. Becher J: *Handleiding Standaard Lichamelijk Onderzoek bij kinderen met een centrale parese,* 1st edn . Reed Business; 2011.

6. American Academy of Orthopedic Surgeons: *Joint Motion: Method of measuring and recording*, 1st edn. E. S. Livingstone, Edinburgh London; 1965.

7. Marshall JL, Girgis FG, Zelko RR: The biceps femoris tendon and its functional significance. *J Bone Joint Surg Am* 1972, 54: 1444-1450.
